# 526. Treatment Inconsistencies of Patients Hospitalized with COVID-19 during Four Waves in Almaty, Kazakhstan, 2020-2021

**DOI:** 10.1093/ofid/ofad500.595

**Published:** 2023-11-27

**Authors:** Saya Gazezova, Roberta Horth, Manar Smagul, Lena Kasabekova, Shokhruh Usmanov, S A N A M ZIKRIYAROVA, Dilyara Nabirova

**Affiliations:** Central Asia Field Epidemiology Training Program, Almaty, Almaty, Kazakhstan; US Centers for Disease Control and Prevention, Dulles, Virginia; Scientific and practical center of sanitary-epidemiological examination and monitoring, branch of the National Center for Public Health, Almaty, Kazakhstan, Almaty, Almaty, Kazakhstan; Central Asia Field Epidemiology Training Program, Almaty, Almaty, Kazakhstan; Central Asia Field Epidemiology Training Program, Almaty, Almaty, Kazakhstan; Kazakh National Medical University named after S.D. Asfendiyarov;, Almaty, Almaty, Kazakhstan; CDC Central Asia office, Almaty, Almaty, Kazakhstan

## Abstract

**Background:**

Clinical management guidelines were continually changing during the COVID-19 pandemic to reflect best available evidence for a novel virus. In Kazakhstan, the treatment guidelines have been modified 16 times since the pandemic began. We assessed compliance with guidelines during four waves of COVID-19 in Kazakhstan.

**Methods:**

We conducted a cross-sectional study among people hospitalized with COVID-19 in an infectious disease hospital in Almaty during 4 waves: T1 (June 1-August 30, 2020); T2 (October 1-31 December 2020); T3 (April 1-May 31, 2021); and T4 (July 1-October 26, 2021). Changes in COVID-19 diagnostic and treatment guidelines published during these periods were studied. We abstracted data from patient electronic medical records.

**Results:**

Seven of 16 COVID-19 diagnostic and treatment guidelines adopted in Kazakhstan were updated during this time. In T1, all people with COVID-19 including those with asymptomatic and mild disease. Antibiotics were recommended only for bacterial infections in T1-T4. Glucocorticosteroids were recommended only to patients with severe disease in T1-T4. Anticoagulants were recommended prophylactically in T1-T3 but only empirically in T4. Of 1,146 COVID-19 patients hospitalized, 14% were in T1, 14% in T2, 22% in T3, and 50% in T4. Mean age was 57 years (range 18-96 years) and 59% were female. In T1-T4 respectively: Antibiotics were given to 78%, 64%, 71%, and 67% of patients with no documentation of bacterial infections; glucocorticosteroids were given to 36%, 31%, 33%, and 28% of patients with non-severe disease; non-COVID-19-specific antivirals were given to 28%, 10%, 4%, and 8% of patients; and anticoagulants given to 18%, 24%, 6%, and 7% of patients with mild disease.

Treatment of hospitalized COVID-19 patients inconsistent with national treatment guidelines, Almaty, Kazakhstan, 2020-2021

**Figure:**
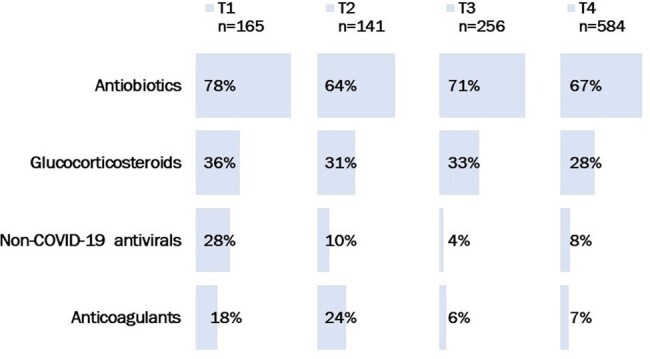

T1: June 1-August 30, 2020; T2: October 1-31 December 2020; T3: April 1-May 31, 2021; T4: July 1-October 26, 2021

**Conclusion:**

We identified inconsistencies with patient treatment and national guidelines. Inappropriate use of antibiotics and non-specific antivirals can result in adverse treatment outcomes and rise in resistance. Improved communication with additional training is needed when guidelines are updated.

**Disclosures:**

**All Authors**: No reported disclosures

